# Factors Influencing Willingness to Pursue Living Kidney Donation Among Relatives of Patients with Kidney Disease in the United States

**DOI:** 10.21203/rs.3.rs-8212449/v1

**Published:** 2025-12-09

**Authors:** Mary K. Roberts, Cayley Ryan-Claytor, Zarmeen Salim, Jennifer M. Kirk, Ian Rowe-Nicholls, Jonathan Daw

**Affiliations:** University of Oxford; University of Michigan–Ann Arbor; Pennsylvania State University; Pennsylvania State University; Pennsylvania State University; Pennsylvania State University

**Keywords:** Living donor kidney transplantation, Donation decision-making, Donor-recipient relationships, Demographic disparities

## Abstract

**Background:**

Living donor kidney transplantation provides superior outcomes for patients with end-stage kidney disease, yet rates of living donation remain low. Identifying factors that influence willingness to donate is essential for developing effective interventions.

**Methods:**

Data were analyzed from 600 U.S. relatives of kidney disease patients in the 2019 Families of Renal Patients Survey. Respondents rated how 14 considerations, including financial, health, and relational factors, affected their willingness to be evaluated as living donors. Associations between each consideration and the number of donation-related actions taken were estimated using Poisson regression models with and without sociodemographic controls.

**Results:**

Improving or extending the recipient’s life and the donor–recipient relationship were the strongest motivators, while concerns about donor health, surgery, and finances were key deterrents. Willingness differed across demographic groups. Older adults were less likely than younger respondents to report reduced willingness for financial reasons, while younger adults more often reported increased willingness based on match likelihood, current health, and knowledge of living donation. Older respondents more often indicated that their own health reduced willingness, and adults aged 40 to 59 most often cited negative effects on their family. Women were more likely than men to report reduced willingness related to financial and recovery concerns. Asian or Pacific Islander and Black respondents more often reported reduced willingness due to financial issues and the surgery itself, while Hispanic and White respondents more often reported increased willingness related to extending the recipient’s life.

Greater willingness related to match likelihood, current health, the donor–recipient relationship, and the potential to extend the recipient’s life was associated with completing more donation-related actions. Lower willingness tied to general views on organ donation, surgery, or recovery was associated with completing fewer actions. After adjustment for demographic factors and relationship to the patient, significant associations remained for match likelihood, current health, the donor-recipient relationship, the potential to extend the recipient’s life, and general views on organ donation.

**Conclusions:**

Interpersonal and health-related considerations were the strongest predictors of donation behavior. Interventions that address these factors may increase willingness to pursue living kidney donation and reduce disparities in access.

## 1. Introduction

Kidney disease remains a major public health challenge in the United States, ranking among the top ten causes of death.^1^ The incidence of end-stage kidney disease (ESKD) has continued to rise, with substantial growth between 2000 and 2022.^2^ Living donor kidney transplantation (LDKT) offers the most effective treatment for ESKD, providing superior survival, quality of life, and graft outcomes compared with dialysis or deceased donor transplantation.^3–7^ Despite these benefits, only 22% of kidney transplants in 2022 were from living donors.^2^ Efforts to expand equitable access to LDKTs are hindered by limited understanding of the factors motivating or discouraging living donation, making it difficult to design effective interventions for those who have never pursued evaluation.

U.S. research has largely focused on patient-, provider-, and system-level barriers to LDKT,^16^ with limited attention to the perspectives of potential donors, particularly those who have not taken concrete steps toward donation. Existing studies often rely on small samples^17,18^ or were conducted outside the U.S. context,^19–21^ limiting generalizability. Prior research on potential living donors has identified multiple motivators and barriers influencing willingness to pursue evaluation.^16,20,21^ These include practical challenges related to the evaluation process,^22^ financial burden,^13,21,24^ concerns about surgical risks and postoperative pain and complications,^8,20^ as well as intra- and interpersonal factors,^16,17,23^ religious beliefs, personal values, and familial or social pressures.^18,20,23,25^

Of these factors that potentially influence donation willingness, financial pressures have received the most attention from both researchers and policymakers. Estimated out-of-pocket and indirect costs for donors range from $2,500 to over $5,000,^26^ encompassing travel, lodging, meals, medical expenses, lost wages, and childcare expenses.^27^ Recent federal initiatives have sought to increase living donation by reducing financial barriers. The National Living Donor Assistance Center (NLDAC) reimburses donors for travel, lodging, and other expenses, while proposed legislation such as the Living Donor Protection Act aims to expand support and ensure paid leave under the Family and Medical Leave Act.^8–11^ Although existing reimbursement programs such as the Living Organ Donation Reimbursement Program^27,28^ aim to offset these costs, assistance is not universally available and often insufficient to fully alleviate the economic burden.^26,29,30^ Moreover, there is little evidence that financial reimbursement programs have significantly increased donation rates.^12–15^

Beyond the limited focus on factors other than financial burdens that influence donation willingness, there is also a limited literature on the ways in which these reasons for or against donation differ across demographic groups.^16,20^ Zazoulina et al. found that while both men and women expressed financial concern, women were more likely to report concerns related to childcare costs.^20^ This is particularly important given that women are more likely to become living donors, comprising approximately 65% of living kidney donors in 2025.^31^

The present study examines factors associated with willingness to undergo evaluation for living kidney donation, focusing on variation by sex, race or ethnicity, and age. Using a large, demographically diverse U.S. sample of adults with a relative affected by kidney disease, this study provides novel insights into potential donor perspectives to inform strategies that promote living donation and reduce disparities in access.

## 2. Materials and Methods

### 2.1 Study Design and Data

This cross-sectional study used data from the second wave of the Families of Renal Patients Survey (FoRPS), a 57-item online survey administered between August 27 and September 1, 2019. FoRPS was developed to assess realistic potential donors’ demographic characteristics, knowledge of living kidney donation, and factors influencing individuals’ willingness and ability to be evaluated as potential living kidney donors. The survey was distributed via the Qualtrics Online Panel (Provo, UT), an established research platform that recruits pre-screened U.S. adults (aged ≥ 18 years) through multiple digital channels including targeted emails, website intercepts, social media, and referral programs. Participants completed the survey online in a non-clinical setting, allowing inclusion of individuals who may not have engaged or otherwise want to engage with the transplant system.

Eligibility criteria included residing in the United States, being 18 years or older, and having at least one relative diagnosed with “weak or failing kidneys.” Respondents with multiple qualifying relatives were instructed to select one individual to consider throughout the survey. Eligible relationships included spouses or partners, parents, children, siblings, grandparents, aunts or uncles, nieces or nephews, cousins, and grandchildren. Friends and other non-family were excluded out of concern that these groups would numerically crowd out fewer common relationships.

Although two survey waves were collected, this analysis is limited to respondents from Wave 2 (n = 600) because this wave included detailed questions about reasons influencing willingness to be evaluated for donation. The study protocol was reviewed and deemed exempt by the Pennsylvania State University Institutional Review Board (Protocol #00005932).

### 2.2 Measures

#### Respondent sociodemographic characteristics

Age was categorized as 18–39, 40–59, and ≥ 60 years. Race and ethnicity were classified as non-Hispanic Asian or Pacific Islander, non-Hispanic Black or African American, non-Hispanic White, Hispanic or Latino/a, and non-Hispanic Other. Sex was coded as male or female. Education is a dichotomous variable indicating whether the respondent holds a bachelor’s degree or higher.

#### Respondent relationship to the patient

Respondents identified patient as their child, parent, sibling, spouse or partner, grandchild, grandparent, niece or nephew, aunt or uncle, cousin, or another relative.

#### Reasons for (un)willingness to donate

The original survey item used to measure reasons influencing respondents (un)willingness to donate to their sick relative was based on extensive prior data collection in a prior online survey with open-ended text questions qualitatively coded by the research team, as described in the Supplemental Section A. In the FoRPS survey, respondents were asked: “When you consider being medically evaluated as a living kidney donor for this person, how did the following factors influence your willingness? Select whether each made you more willing, less willing, or neither more nor less willing to do so.” The 14 considerations were: financial reasons; likelihood of being a match; current health; knowledge of living kidney donation; relationship with the patient; moral, religious, or ethical reasons; impact on the rest of the family; general views on organ donation; extending the patient’s life; impact on own health; kidney donation surgery; improving the patient’s quality of life; post-surgical recovery; and other characteristics of the patient. Respondents could indicate whether each factor made them more willing to donate, less willing to donate, or neither willing nor less willing to donate.

#### Living donation actions

A cumulative measure ranging from 0 to 5 counted the number of concrete actions respondents reported taking toward donation: (1) discussing donation with the patient; (2) agreeing to be medically evaluated; (3) undergoing medical evaluation; (4) being medically approved as a potential donor; and (5) donating a kidney.

### 2.2 Statistical Analysis

We first generated descriptive statistics of willingness by reason for the full sample and stratified by age, sex, and race or ethnicity. Chi-squared tests assessed whether distributions across willingness categories differed significantly by demographic subgroup.

We then estimated a series of Poisson regression models to assess associations between willingness for each reason and the number of living donation actions taken, as this outcome represents a non-negative count of discrete behaviors toward living donation. Results are presented as average marginal effects, representing the average change in the number of donation steps taken relative to the reference category (“neither more nor less willing”). Both unadjusted and adjusted models were estimated, with controls for age, education, sex, and relationship to the patient.

To improve representativeness, post-stratification weights were applied to align the demographic characteristics of the reported relatives with those of the national transplant candidate population, based on data from the 2016 U.S. Renal Data System. Analyses were conducted using Stata/SE version 18.0 (StataCorp LLC, College Station, TX).

## 3. Results

### 3.1 Descriptive statistics

After applying poststratification weights, most respondents were between 18 and 39 years old (54%), while 35% were aged 40 to 59 and 10% were 60 or older. Approximately 63% of participants were female, and 32% held a bachelor’s degree or higher. The weighted sample was 46% non-Hispanic White, 26% non-Hispanic Black or African American, 17% Hispanic or Latino/a, 5% non-Hispanic Asian or Pacific Islander, and 4% identified as another race or ethnicity. The most common patient–donor relationship was parent (27.7%), followed by aunt or uncle (17%), spouse or partner (16%), and grandparent (15%).

**Figure 1:**
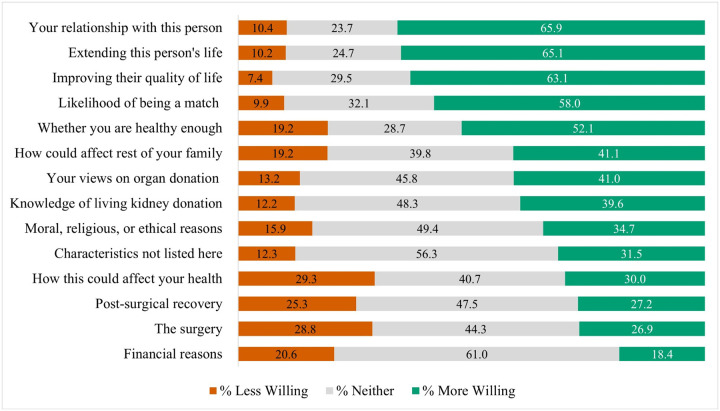
Living Kidney Donation Willingness by Reasons

Figure 1 summarizes the distribution of reasons related to willingness to pursue living donor kidney transplantation. Four reasons were most widely endorsed as motivators: the respondent’s relationship with the recipient (65.9% more likely), extending the recipient’s life (65.1%), improving their quality of life (63.1% more likely), and the likelihood of being a match (58.0%). These were far more often reported as increasing rather than decreasing willingness to donate (≤ 10% marked less willing for these items).

The most common deterrents were concerns about the donor’s own health (29.3%), the kidney donation surgery (28.8%), post-surgical recovery (25.3%), and financial factors (20.6%). However, in each of these cases, comparable or greater proportions of respondents also indicated that these factors made them more willing to donate. Full distributions for all reasons are provided in Supplemental Table B.

Table 2 presents differences in willingness to donate by age group. Distributions across reasons differed significantly by age for six factors: financial reasons, likelihood of being a match, current health, knowledge of living kidney donation, effects on the rest of the family, and potential effects on personal health (p < 0.05). Compared with respondents aged 18–39 (22.4%) or 40–59 (19.0%), those aged ≥ 60 (16.5%) were significantly less likely to report financial reasons as making them less willing to donate. Respondents aged 18–39 (61.1%) were more likely to report that their likelihood of being a match made them more willing to donate compared with those aged 40–59 (57.2%) or ≥ 60 (44.4%). Younger respondents (aged 18–39) were also more likely to report that their current health made them more willing to donate (68.6%), whereas those aged ≥ 60 (30.2%) were more likely to report it made them less willing. Respondents aged 18–39 (44.4%) were more likely than older groups to indicate that their knowledge of living kidney donation increased their willingness to donate. Those aged 40–59 (42.6%) were more likely to cite potential effects on the rest of their family as making them less willing to donate compared with those aged 18–39 (15.7%) or ≥ 60 (19.0%). Finally, respondents aged ≥ 60 (32.4%) were more likely to report that potential effects on their own health made them less willing to donate compared with those aged 40–59 (31.4%) or 18–39 (25.3%).

As shown in Table 3, willingness also differed significantly by sex for two reasons. Women were more likely than men to report decreased willingness when considering financial factors (24.8% vs. 13.5%, p < .05) and post-surgical recovery (28.9% vs. 19.2%, p < .05).

Table 4 presents differences in willingness to donate by race and ethnicity. When considering financial reasons, 28.7% of Asian or Pacific Islander and 30.0% of Black or African American respondents reported being less willing to undergo LDKT, compared with 18.3% of White and 5.2% of Hispanic respondents (p < 0.01). Hispanic respondents were also more likely than other groups to report that financial considerations made them more willing to consider donation. For the factor extending the recipient’s life, Hispanic (71.7%) and White (67.1%) respondents were more likely to report increased willingness compared with Black (58.2%) and Asian or Pacific Islander (55.9%) respondents, while White respondents were less likely to report decreased willingness (p < 0.05). A greater proportion of Asian or Pacific Islander (32.1%) and Black (44.6%) respondents reported being less willing to donate when considering the surgery itself, compared with White (24.8%) and Hispanic (9.4%) respondents (p < 0.05).

### 3.2 Regression analysis

**Figure 2:**
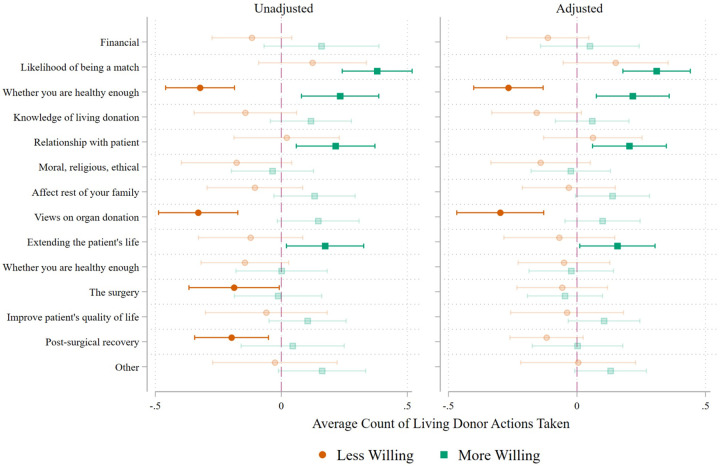
Average Marginal Effects of Number of LDKT Actions Taken

Figure 2 presents the estimated average marginal effects for each reason on the number of living donation actions taken, both unadjusted and adjusted for sociodemographic characteristics. The “neither more nor less willing” category served as the reference group for all models. Supplemental Table C contains the corresponding coefficients.

Several reasons were significantly associated with the number of actions taken toward donation, and all statistically significant associations were in the expected direction - i.e., respondents who were less willing to consider donation due to a specific factor completed fewer donation-related actions, whereas those who were more willing completed more.

Prior to adjustment for respondent age, sex, race/ethnicity, education, and relationship to the patient, respondents who reported that their current health made them less willing to donate completed 0.32 fewer steps toward donation (p < 0.01) compared with those who reported being “neither less nor more willing.” Conversely, respondents who indicated that their current health made them more willing to donate completed 0.23 more steps toward donation (p < 0.01). Respondents who reported that their likelihood of being a match (AME = 0.38, p < 0.01), their relationship with the patient (AME = 0.22, p < 0.01), and the potential to extend the patient’s life (AME = 0.17, p < 0.05) made them more willing to donate also completed more actions toward donation, on average. Finally, respondents who indicated that their views on organ donation (AME = −0.33, p < 0.01), concerns about post-surgical recovery (AME = −0.20, p < 0.01), and apprehension regarding the donation surgery itself (AME = −0.19, p < 0.05) made them less willing to donate completed fewer actions toward donation, on average.

In the adjusted model, respondents who indicated that being healthy made them more willing to consider donation completed, on average, 0.22 more actions (p < 0.01), whereas those who reported being less willing because of health concerns completed 0.27 fewer actions (p < 0.01). Respondents who were more willing due to the likelihood of being a match completed 0.31 more actions (p < 0.01), and those whose willingness increased because of their relationship with the recipient completed 0.20 more actions (p < 0.01). Greater willingness associated with the potential to extend the recipient’s life (AME = 0.16, p < 0.05) also predicted more actions taken. In contrast, respondents whose general views on organ donation made them less willing to donate completed 0.30 fewer actions on average (p = 0.01). Concerns regarding post-surgical recovery and the donation surgery itself were no longer statistically significant after adjustment for respondent demographics and relationship to the patient.

## 4. Discussion

The number of individuals added to the kidney transplant waitlist each year is nearly six times higher than the number of living donor kidney transplantations performed.^32^ Understanding the factors that shape willingness to consider donation is therefore critical for developing interventions that can increase living donor kidney transplantation. Despite this importance, research on potential donors’ perspectives remains limited, particularly among those who take no actions toward living donation. Previous studies have often relied on small or nonrepresentative samples or have been conducted outside the United States, reducing their relevance to U.S. populations.^33,34^ The present study addresses this gap by using a large, nationally distributed sample of realistic potential living donors and by directly examining how specific reasons influence both willingness to donate and concrete donation-related actions.

Our findings indicate that interpersonal and health-related factors are the most salient influences on willingness to pursue living donation and the strongest predictors of action. Reasons related to the respondent’s relationship to the patient, the perceived likelihood of being a match, and confidence in personal health were consistently associated with taking more concrete steps toward donation. In contrast, although concerns about surgery, recovery, and financial cost were commonly cited as deterrents, they did not consistently predict donation behavior after adjusting for sociodemographic factors. These results suggest that while such concerns are widespread, they may not be the primary barriers preventing potential donors from progressing through the donation process.^27,28^

These findings have important implications for ongoing policy efforts to increase living donation. Federal initiatives such as the NLDAC and proposed legislation, including the Living Donor Protection Act, primarily focus on reducing the financial burden of donation.^12,14,27,28,35^ The NLDAC provides reimbursement for travel, lodging, dependent care, and lost wages to reduce economic disincentives for eligible donors, while the Living Donor Protection Act seeks to prohibit discrimination in life, disability, and long-term care insurance based on donor status and to ensure coverage under the Family and Medical Leave Act.^8,9,11^ Additional legislative proposals, such as the Honor Our Living Donors Act and the Living Organ Donor Tax Credit Act, aim to expand NLDAC eligibility by focusing on donor rather than recipient income and to provide tax credits for unreimbursed donation-related expenses.^10,36^ Collectively, these programs have expanded access to reimbursement for travel, lodging, dependent care, and lost wages, while also seeking to ensure job and insurance protections.^37^ Although these initiatives represent essential progress, our findings suggest that focusing primarily on financial incentives may be insufficient,^12,15^ indicating that interpersonal and health-related considerations remain stronger predictors of donor action. Potential donors appear to be more strongly motivated by their relationship with the recipient, their perceived health, and the anticipated benefits for the recipient and family than by economic considerations alone. Future intervention efforts should focus on addressing these topics as well as removing financial disincentives to donation.

Given the breadth of reasons affecting substantial shares of potential donors, policies should instead be part of a broader framework that also strengthens education, psychosocial support, and public awareness. Expansion of existing programs to include counseling, navigation services, and family-inclusive education could help address the interpersonal and health-related factors identified in this study. Ensuring that information about financial and non-financial supports is easily accessible through primary care, dialysis centers, and transplant clinics may improve reach and equity.^38^ State-level policies that provide tax credits, paid leave, or travel stipends could further complement federal initiatives, but these too must be paired with communication strategies that emphasize safety, follow-up care, and long-term well-being.

The clinical implications of these findings are equally significant. Transplant teams, coordinators, and donor advocates play central roles in shaping how potential donors understand the risks and benefits of living donation.^39^ Our results suggest that programs should prioritize personalized and family-centered approaches to donor counseling. Because the donor–recipient relationship was the strongest motivator identified, early inclusion of family members in discussions may strengthen donor confidence and reduce ambivalence. Structured family-based counseling could also help donors articulate their motivations and boundaries, minimizing the risk of perceived pressure.

Concerns about health, surgery, and recovery underscore the importance of transparent communication about medical evaluation, surgical procedures, and long-term outcomes. Donors should receive clear, evidence-based information about perioperative safety, potential complications, and recovery trajectories. Incorporating former donors as peer navigators or mentors could provide reassurance through shared experience and may increase trust among hesitant individuals. In addition, transplant programs could implement pre-donation decision aids that address both practical and emotional considerations. Tailoring these materials to reflect cultural and linguistic diversity may further enhance engagement among underrepresented groups.

We also observed meaningful differences by demographic characteristics. Women, Black and Asian respondents, and older adults were more likely to report reduced willingness when considering financial and surgical concerns. These differences underscore the need for tailored approaches that address the specific concerns of diverse potential donor populations. For example, discussing available financial protections may be particularly important for older or lower-income donors, while ensuring clear communication about surgical risk and recovery may be critical for groups expressing greater health-related apprehension. Personalized education and counseling that account for demographic context may improve both willingness and follow-through among potential donors. Addressing these inequities requires culturally responsive outreach, partnerships with community and faith-based organizations, and messaging that speaks to both the risks and the transformative potential of donation.^40^ Transplant education campaigns should be designed collaboratively with communities to reflect their values, experiences, and concerns.

This study has several limitations. The data are cross-sectional and based on self-reported measures, which precludes causal inference and may not capture changes in donor decision-making over time. The sample, although weighted to approximate the national transplant candidate population, was recruited online and may underrepresent individuals without internet access or those less likely to participate in survey research. Additionally, willingness to be evaluated does not necessarily translate into actual donation, and factors influencing donation behaviors may differ at different stages of the process. Despite these limitations, the study provides novel, generalizable insights into the early stages of donor decision-making in a U.S. population.

In sum, our study of a sample of realistic potential kidney donors in the U.S. reveals the importance of interpersonal and health factors in influencing both the willingness to donate and actions taken towards donation. Our analyses show that only certain donation considerations were both salient to respondents and significantly associated with the number of steps they took toward donation: respondents’ likelihood of being a match, their views on organ donation, their relationship to patient, and the potential effects of donation on the rest of the family. Current policies that specifically target economic costs as the primary barrier to donation as well as one-sized-fits-all approaches to discussions with potential donors are unlikely to be successful as interventions that address potential donors’ interpersonal, relational, and health considerations.

## Supplementary Material

Supplementary Files

This is a list of supplementary files associated with this preprint. Click to download.

- Supplementary.docx

## Figures and Tables

**Table 1:** Unweighted and Weighted Sample Characteristics

	Unweighted	Weighted
**N**	600	600
**Respondent Age**		
18–39	55.67	54.37
40–59	32.67	35.26
60+	11.67	10.38
**Respondent Sex**		
Female	61.67	63.12
Male	38.33	36.88
**Respondent Education**		
<BA	68.50	67.72
BA+	31.50	32.28
**Respondent Race/Ethnicity**		
NH-Asian or Pacific Islander	5.17	5.43
NH-Black or African-American	16.50	26.58
NH-Caucasian or White	64.83	46.31
Hispanic or Latino/a	9.00	17.62
Other	4.50	4.06
**Relationship to Patient**		
Child	2.50	0.95
Parent	26.83	27.70
Sibling	7.50	7.39
Spouse/Partner	20.33	16.56
Grandchild	0.17	0.04
Grandparent	14.67	14.91
Niece/Nephew	1.00	0.98
Aunt/Uncle	14.33	17.57
Cousin	5.67	5.94
Other	7.00	7.96

Note: NH = non-Hispanic

**Table 2:** Weighted Distribution of Reasons for and Against Living Kidney Donation Willingness by Respondent Age.

	18–39		40–59		60+		
	(N = 334)		(N = 196)		(N = 70)		
Reasons	% Less Willing	% More Willing	% Less Willing	% More Willing	% Less Willing	% More Willing	p-value
Financial reasons	22.42	20.60	19.03	18.96	16.53	4.48	0.02
Likelihood of being a match	10.71	61.08	8.35	57.24	10.57	44.37	0.02
Whether you are healthy enough	16.42	57.43	20.27	49.68	30.24	32.62	0.00
Knowledge of living kidney donation	12.75	44.38	12.24	35.16	8.83	29.13	0.04
Your relationship with this person	10.87	68.89	6.38	65.06	21.85	52.57	0.09
Moral, religious, or ethical reasons	16.24	34.95	11.84	38.54	27.92	20.17	0.22
How could affect rest of your family	15.73	43.32	24.28	42.59	19.78	24.21	0.00
Your views on organ donation	11.10	43.46	14.96	43.67	18.52	18.78	0.06
Extending this person’s life	8.18	69.01	11.85	62.84	15.36	52.20	0.24
How could affect your health	25.34	32.23	31.37	29.23	42.85	20.74	0.01
The surgery	25.71	30.13	29.90	23.80	41.25	20.78	0.45
Improving their quality of life	8.92	64.25	5.55	63.90	5.34	54.33	0.15
Post-surgical recovery	23.81	30.51	25.12	24.91	33.75	17.57	0.48
Characteristics not listed above	13.23	32.93	11.98	31.93	8.23	22.20	0.59

NOTE: Percentages of sample in each category are weighted to match the demographic characteristics of the U.S. ESKD patient population. Chi-squared tests are based on unweighted values. Bolded and underlined chi-squared values indicate statistical significance at the p ≤ 0.05 level.

**Table 3:** Distribution of Reasons for and Against Living Kidney Donation Willingness by Sex.

	Female		Male		
	(N = 370)		(N = 230)		
Reasons	% Less Willing	% More Willing	% Less Willing	% More Willing	p-value
Financial reasons	24.78	17.40	13.48	19.98	0.04
Likelihood of being a match	12.23	54.96	5.81	63.18	0.25
Whether you are healthy enough	22.09	52.04	14.29	52.25	0.43
Knowledge of living kidney donation	12.24	41.58	12.04	36.07	0.42
Your relationship with this person	10.31	65.47	10.64	66.49	0.91
Moral, religious, or ethical reasons	15.17	36.40	17.17	31.74	0.43
How could affect rest of your family	21.71	43.66	14.81	36.68	0.14
Your views on organ donation	13.12	42.80	13.42	37.85	0.69
Extending this person’s life	10.33	65.28	10.03	64.75	0.64
How could affect your health	32.03	30.67	24.59	28.79	0.21
The surgery	33.82	25.26	20.20	29.78	0.08
Improving their quality of life	8.07	64.52	6.16	60.67	0.46
Post-surgical recovery	28.88	29.30	19.17	23.59	0.05
Characteristics not listed above	13.16	33.11	10.74	28.65	0.61

NOTE: Percentages of sample in each category are weighted to match the demographic characteristics of the U.S. ESKD patient population. Chi-squared tests are based on unweighted values. Bolded and underlined chi-squared values indicate statistical significance at the p ≤ 0.05 level.

**Table 4:** Distribution of Reasons for and Against Living Kidney Donation Willingness by Race/Ethnicity.

	NH-Asian or Pacific Islander		NH- Black or African-American		NH-Caucasian or White		Hispanic or Latino/a		
	(N = 31)		(N = 99)		(N = 389)		(N = 54)		
Reasons	% Less Willing	% More Willing	% Less Willing	% More Willing	% Less Willing	% More Willing	% Less Willing	% More Willing	p-value
Financial reasons	28.72	7.16	30.02	18.15	18.29	16.32	5.15	30.90	0.00
Likelihood of being a match	15.44	50.72	9.98	59.27	5.75	60.62	17.02	50.88	0.14
Whether you are healthy enough	22.64	46.03	23.07	49.70	14.87	55.73	14.96	54.91	0.66
Knowledge of living kidney donation	19.90	22.78	18.40	36.30	7.87	41.14	6.99	44.51	0.06
Your relationship with this person	28.01	53.21	12.21	70.39	6.00	67.62	10.22	58.27	0.07
Moral, religious, or ethical reasons	23.17	24.93	18.16	41.78	13.16	31.26	12.95	41.59	0.29
How could affect rest of your family	27.30	29.47	22.61	46.03	13.99	38.70	22.70	43.73	0.35
Your views on organ donation	13.51	53.49	18.27	43.02	8.72	39.60	16.37	36.71	0.18
Extending this person’s life	22.66	55.90	11.89	58.24	5.23	67.10	15.57	71.74	0.03
How could affect your health	40.34	22.23	36.85	28.11	25.94	27.58	20.45	38.02	0.39
The surgery	32.12	20.66	44.60	26.55	24.80	26.52	9.35	34.55	0.04
Improving their quality of life	11.97	54.06	11.43	66.02	4.27	66.32	5.55	51.50	0.03
Post-surgical recovery	23.00	22.65	31.90	24.73	21.98	27.10	15.87	37.29	0.59
Characteristics not listed above	8.91	19.65	15.39	37.84	7.52	29.53	16.27	35.25	0.04

NOTE: Percentages of sample in each category are weighted to match the demographic characteristics of the U.S. ESKD patient population. Chi-squared tests are based on unweighted values. Bolded and underlined chi-squared values indicate statistical significance at the p ≤ 0.05 level.

## Data Availability

The datasets generated and/or analysed during the current study are available from the corresponding author on reasonable request.

## Abbreviations

AME: Average Marginal Effect
ESKD: End–Stage Kidney Disease
FoRPS: Families of Renal Patients Survey
LDKT: Living Donor Kidney Transplantation
NLDAC: The National Living Donor Assistance Center
